# The Use of Phototherapy for the Treatment of Non-Seasonal Depression: A Systematic Review of Efficacy and Safety

**DOI:** 10.3390/jcm14051756

**Published:** 2025-03-05

**Authors:** Andrei Lomnasan, Bogdan Ioan Vintilă, Mihaela Bucuța, Laura Ștef, Claudia Elena Anghel, Andreea Maria Grama, Monica Cornea, Adrian Boicean, Cristian Ichim, Lucian Constantin Paziuc, Mihnea Costin Manea, Andrian Tîbîrnă, Ciprian-Ionuț Băcilă

**Affiliations:** 1 “Dr. Gheorghe Preda” Clinical Psychiatry Hospital of Sibiu, 550082 Sibiu, Romania; andreilomnasan96@gmail.com (A.L.); claudia.anghel@ulbsibiu.ro (C.E.A.); andreeamariaps@gmail.com (A.M.G.); moni.sipos@ymail.com (M.C.); ciprian.bacila@ulbsibiu.ro (C.-I.B.); 2 Faculty of Medicine, Lucian Blaga University of Sibiu, 550169 Sibiu, Romania; laura.stef@ulbsibiu.ro (L.Ș.); adrian.boicean@ulbsibiu.ro (A.B.); cristian.ichim@ulbsibiu.ro (C.I.); 3 County Clinical Emergency Hospital of Sibiu, 550245 Sibiu, Romania; 4 Neuroscience Scientific Research Collective, 550082 Sibiu, Romania; 5 Faculty of Social Sciences and Humanities, Lucian Blaga University of Sibiu, 550024 Sibiu, Romania; 6 Campulung Moldovenesc Psychiatric Hospital, Trandafirilor Street 2, 725100 Câmpulung Moldovenesc, Romania; paziuc.lucian@yahoo.com; 7 “Prof. Dr. Alexandru Obregia” Clinical Hospital of Psychiatry, 041914 Bucharest, Romania; mihnea.manea@umfcd.ro (M.C.M.); atibirna@yahoo.com (A.T.); 8 Faculty of Medicine, Carol Davila University of Medicine and Pharmacy, 8 Eroii Sanitari Bvd, 050474 Bucharest, Romania

**Keywords:** phototherapy, non-seasonal depression, treatment efficacy, safety profile

## Abstract

**Background**: Phototherapy, which has traditionally been used for seasonal affective disorder, is now being investigated for its effectiveness in treating non-seasonal depression. This systematic review aims to evaluate the efficacy and safety of phototherapy in this new context, providing a comprehensive overview of its therapeutic potential and limitations. **Methods**: The review followed PRISMA guidelines and included studies from databases such as Google Scholar, PubMed, and UpToDate. Studies were selected based on their focus on phototherapy’s efficacy, safety, and application methods for non-seasonal depression. Various administration methods were examined, particularly the effects of multiple daily sessions and personalized treatment plans. **Results**: The findings indicate that while phototherapy alone has limited effectiveness, combining it with antidepressants significantly improves outcomes. The most effective protocols featured multiple daily sessions tailored to individual patient needs, even at lower light intensities. Safety assessments have shown that phototherapy is well tolerated, with no serious side effects reported, only minor and transient reactions. **Conclusions**: Phototherapy appears to be a promising adjunct therapy for non-seasonal depression, offering safety and flexibility in treatment customization. It provides consistent therapeutic benefits, mainly when used in conjunction with conventional antidepressant treatments.

## 1. Introduction

Phototherapy is a treatment method for depression that can be used both alone and in combination with pharmacological treatment, being a method suitable for both children and adults. In addition to depression, phototherapy is also a method for treating sleep disorders [1,2,3,4,5].

Phototherapy is indicated for the treatment of both seasonal and non-seasonal depression [6]; other indications include the treatment of sleep disorders and bipolar depression [1]. The light therapy is usually conducted using a light intensity of 10,000 lux, and each session typically lasts 30 min. However, there are different specific application methods, such as 2500 lux for a period of two hours [1,6,7,8]. Morning exposure is generally recommended, as it aligns with the body’s natural circadian rhythms and can help regulate melatonin production. Despite its effectiveness, factors such as cost, access to quality light therapy devices, and patient adherence to treatment schedules influence its prevalence of use [1,6,7,8].

While the exact mechanism of action remains incompletely understood, several hypotheses have been proposed. One prominent theory suggests that phototherapy modulates serotonin activity in the brain, which is crucial for regulating mood. Another hypothesis states that exposure to bright light inhibits melatonin secretion, thereby reducing daytime sleepiness and enhancing alertness. Additionally, phototherapy may help synchronize the circadian rhythm, which can become disrupted in individuals with depression. By re-aligning the body’s internal clock, phototherapy can contribute to improved mood and overall well-being [9].

Phototherapy is considered a safe procedure without adverse reactions, but isolated cases have reported effects such as diarrhea, headache, and palpitations [9]. Other adverse reactions may include blurred vision and eye irritation; however, these reactions do not persist in the long term [1,10,11]. Phototherapy can also induce a shift towards a manic episode, especially in patients with mood swings; however, the risk of inducing mania is very low [1,12].

The best time of day for phototherapy is in the morning, after waking up. According to data from the literature, after phototherapy, approximately 60% to 90% of patients recover from seasonal depression; in this case, phototherapy is used daily in autumn and winter. In contrast, for non-seasonal depression, it is used continuously until symptom remission, including in spring and summer. When used as a standalone treatment method, the remission rate is 44%. However, according to specialized data, the remission rate increases to 59% when combined with fluoxetine and 76% when combined with venlafaxine [1,2,13].

Phototherapy devices can use various colors, with blue and white light most commonly used [14]. The phototherapy device consists of a specialized light box that emits light with adjustable intensity parameters. Patients sit in front of the light source during treatment for 30 to 60 min each day, ideally in the morning, to help synchronize their circadian rhythms. The light box is positioned at an angle that allows the light to reach the eyes indirectly, so patients do not need to stare directly into the light. Instead, they can engage in daily activities such as reading, eating, or working while remaining near the device. This design facilitates sufficient exposure to therapeutic light without disrupting the patient’s routine [14].

This study aims to assess the efficacy and safety of phototherapy in the treatment of unipolar non-seasonal depression. We also aim to analyze the application of phototherapy, whether it is effective as an augmentation method to pharmacological therapy or as a standalone treatment method. Regarding safety, we will analyze which adverse reactions have been identified and whether they had a long-term impact, and we will evaluate the safety of this method.

## 2. Materials and Methods

The review was conducted according to PRISMA criteria, applying specific exclusion and inclusion criteria to select the studies.

We analyzed 30 studies from Google Scholar, PubMed, and UpToDate platforms. Of the 30 studies analyzed, 16 were included, and 14 were excluded.

The inclusion criteria consisted of studies that addressed phototherapy in unipolar non-seasonal depression and studies that focused on topics such as efficacy, safety, and combination with other depression treatment methods. Studies that addressed phototherapy as a treatment method for bipolar depression, studies that focused exclusively on phototherapy as a treatment for seasonal depression, and case presentations were excluded. The studies included original research, meta-analyses, and systematic reviews.

The keywords used were non-seasonal depression, phototherapy, efficacy, augmentation, adverse effects, lux, duration, white light, blue light, green light, and seasonal depression.

After analyzing the studies, relevant results for our review were selected and extracted. Then, these data were analyzed, interpreted, and correlated to create the most objective view of phototherapy. The aim was to determine the effect of phototherapy on depression, the impact of phototherapy in combination with antidepressant medication, and identify possible adverse reactions.

## 3. Results

**Figure 1:**
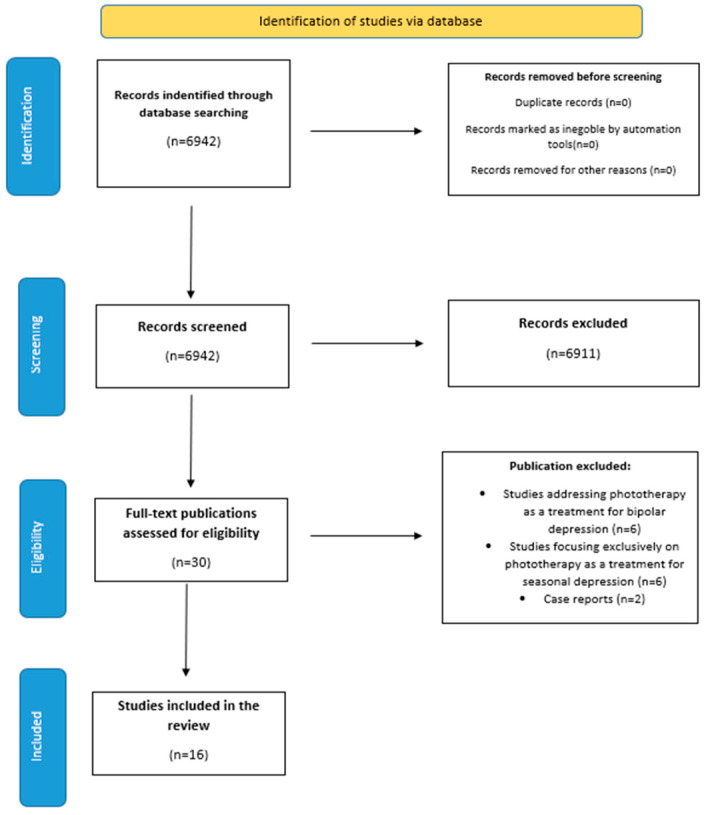
PRISMA flow diagram.

**Figure 2:**
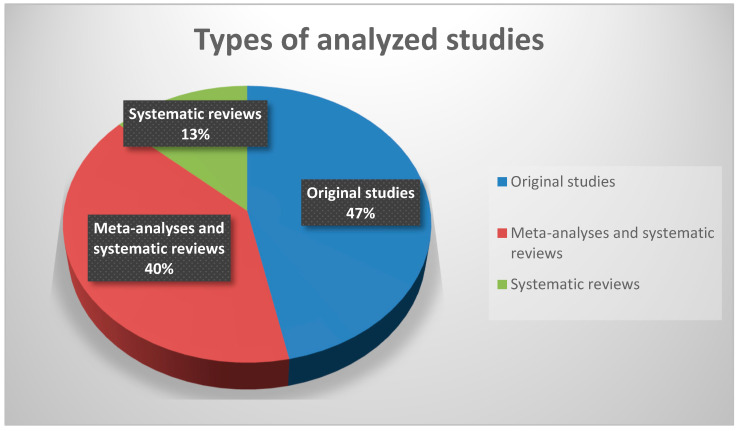
Types of analyzed studies.

A total of n = 16 studies were analyzed; the analyzed studies are presented in Table 1. Of the analyzed studies (n = 16), 7 were original, 6 were meta-analyses and systematic reviews, and 3 were systematic reviews (Figure 1). The studies included in the analysis and their characteristics are presented in Table 1 and Table 2 and Figure 2.

### 3.1. Efficacy of Phototherapy

To evaluate the efficacy of phototherapy, we analyzed studies that compared the effects of light therapy on both seasonal and non-seasonal depression. We also examined studies that investigated the effects of phototherapy on patients with other psychiatric comorbidities, such as psychosomatic patients and those with underlying personality disorders.

The exact mechanism of phototherapy is not well understood. Still, exposure to ultraviolet light has been observed to improve the well-being of patients with depression and lead to a reduction in depressive symptoms [29].

The study conducted by Boghos I. Yerevanian, Janis L. Anderson, Lee J. Grota, and Marjorie Bray showed that phototherapy has therapeutic effects on seasonal depression. Seven out of nine patients showed an improvement in symptoms. Still, it is ineffective in non-seasonal depression, with none of the eight patients responding to phototherapy [30].

However, it has been shown that phototherapy can have beneficial effects on non-seasonal depression, not as a standalone treatment but rather in combination with antidepressant medication [18,19,31]. Several studies which were analyzed support the idea of combining phototherapy with medication. In a study by Funda Güdücü, Okan Çaliyurt, Erdal Vardar, Cengiz Tuğlu, and Ercan Abay, it was demonstrated that sertraline combined with phototherapy reduces the intensity of anxiety and depression so that by the end of the study, 38.1% of patients showed a decrease in symptom intensity, measured by HAMILTON scale scores [20]. The improvement in symptoms through combining phototherapy with antidepressant medication can be explained by the synergistic effect of these two treatment methods [15].

A study evaluating the importance of the ultraviolet radiation spectrum in phototherapy found that 20.8% of participants showed symptom improvement after two weeks. After four weeks, only 8% of participants still had symptoms. The maximum effect of phototherapy was evident in the first 4 weeks [27].

Phototherapy has also been found effective in treating sleep disorders. Following phototherapy sessions, improved sleep quality was observed [22,23]. Specifically, exposure to light therapy in the evening reduces daytime sleepiness and improves nighttime sleep quality [22]. Another method for reducing daytime sleepiness through phototherapy has been found to be morning sessions [15]. Phototherapy may also have applicability in somatizations; after 4 weeks of phototherapy, a reduction in headache was observed, but there was also an aggravation of vertigo, with the exact mechanism not being explained [22].

A limitation of phototherapy is that it does not affect all types of depressive symptoms, affecting typical symptoms but not atypical ones [21].

However, the effect of phototherapy on non-seasonal depression is considered controversial. A meta-analysis and systematic review study found that phototherapy has a low to moderate impact on the remission of depressive symptoms. A more favorable effect was observed in patients receiving phototherapy on an outpatient basis compared to those hospitalized [25,32]. The same study suggests that light therapy might be more effective if used as a standalone treatment method for low-severity depression [25,32].

Phototherapy has also proven effective in elderly patients with non-seasonal depression. It was found that the severity of depressive symptoms decreased after phototherapy, and there were no physical adverse reactions, with none of the patients experiencing manic episodes following phototherapy [16,33].

Another factor considered in the analyzed studies was the effect of phototherapy on non-seasonal depression in patients with other psychiatric comorbidities. The review included two studies that examined this topic: one study explored the effect of phototherapy on psychosomatic patients, and another study investigated the impact of phototherapy in patients with non-seasonal depression and borderline personality disorder.

The article by Tayebmanesh L and Saadati N demonstrates that phototherapy, combined with occupational therapy, improves the quality of life of psychosomatic patients, reducing anxiety in 10% of patients and depression in 22% of patients [28].

One of the studies included in the review concluded that phototherapy, combined with antidepressant medication, has a beneficial effect on improving symptoms in patients with treatment-resistant non-seasonal depression and borderline personality disorder, and it was considered that this combination might also be used in cases of resistance to antidepressant treatment [26]. After 6 weeks, it was found that depressive symptoms improved, and patients no longer had self-harming tendencies. However, two patients began to have self-harming tendencies again 2 weeks after the end of phototherapy sessions [26]. The severity of symptoms was measured using various scales such as HAM-D, MARDS, and BDI; these scales were applied weekly over 6 weeks, and it was found that the scores decreased during these 6 weeks [26]. Additionally, it was observed that patients no longer exhibited impulsivity, self-harming tendencies, or feelings of inner emptiness [26].

### 3.2. Application of Phototherapy

In this subsection, we will analyze the application of phototherapy based on the type of light used, light intensity, number of sessions per day, and the time of day when phototherapy sessions are administered. The goal is to identify whether there are significant differences in the effect of phototherapy on depression depending on the color of the light, its intensity, and the timing of phototherapy throughout the day. We will also examine whether multiple daily sessions improve patient outcomes compared to a single session and, if so, how many sessions are necessary.

Phototherapy can be administered using a range of light colors, predominantly white, blue, and green, with studies showing that these are equally effective regardless of the type of light used [15,16,17,34,35]. Blue light’s efficacy in treating seasonal and non-seasonal depression remains unproven [17]. Another study evaluated the effect of red light on depression and concluded that it does not affect depressive manifestations [21].

Generally, phototherapy is administered in a single session after waking up. Studies have shown that administering phototherapy both in the morning and evening may be more effective than a single session per day [21].

In a study by Mohammad A. Alotaibi, Mark Halaki, and Chin-Moi Chow, several studies were analyzed regarding the application method, and it was observed that the session duration varies between 30 min and 2 h [15]. In 1 of the studies analyzed, sessions were conducted in the morning, at noon, or in the evening, while in the majority of studies, phototherapy was performed exclusively in the morning; 3 out of 17 studies did not demonstrate favorable effects when phototherapy was used in the morning [15].

The time of day when phototherapy is applied can influence the circadian rhythm. Applying phototherapy in the evening has been shown to improve nighttime sleep quality and reduce daytime sleepiness [22]. Regarding daytime sleepiness, another study supports that light therapy should be applied in the morning to reduce it [15].

Another important aspect is light intensity, measured in lux. A standard phototherapy session uses light at an intensity of 10,000 lux for 30 min [1,6,7].

However, various methods of applying light intensity in phototherapy exist. One study demonstrated that an intensity of over 5000 lux and a session duration of at least 30 min enhance the effect of antidepressant medication [24].

In a systematic review analyzing the duration, type of light, and light intensity, several studies showed that positive effects appear at light intensities between 4000 and 10,000 lux [15]. A total of 79% of the analyzed studies indicate that light exposure therapy has a positive impact on the treatment of depression [15]. Studies have shown that a high light intensity is not mandatory, but lower intensities, such as 176, 400, 1000, or 1500 lux, are also sufficient to alleviate depressive symptoms [15].

These studies show that different phototherapy methods are used based on intensity, light type, number of sessions, and timing throughout the day.

Regarding light intensity and color, no significant differences were identified, with results being similar regardless of the color type, and both low and high intensities had a positive effect. Concerning the number of sessions, it is observed that applying two sessions per day is more effective than a single session. As for the time of day, phototherapy is most often used in the morning; another adequate time is in the evening, especially if phototherapy is administered twice a day, first in the morning and again in the evening, but there are also studies where phototherapy was applied at noon.

### 3.3. Safety of Phototherapy

In this subsection, we will present the results from studies regarding adverse reactions that may occur with phototherapy, considering whether they are transient or have a long-term impact. Based on the identified data, we will determine the safety of phototherapy at the end of this analysis.

Studies have found that the most common adverse reactions are agitation, sleep disturbances, palpitations, and irritation of the eye globes [19]. These adverse reactions are rare and tend to be transient and short-term, without significant impact or long-term discomfort for the patient [19]. Another theoretical adverse reaction is the shift towards a manic episode; in the analyzed study, only one patient experienced this reaction [14].

Phototherapy is a safe treatment method even for elderly patients; geriatric patients did not exhibit physical or psychological adverse reactions, and none of the patients developed a manic episode [16,34,35].

It was observed that light intensity does not influence adverse reactions [22].

Phototherapy does not have absolute contraindications. The contraindications relate to patients with ophthalmological diseases and are considered more preventive recommendations rather than strict contraindications [19].

Overall, phototherapy is a safe treatment method with rare and short-lived adverse reactions and is safe for elderly patients.

## 4. Discussion

The effect of phototherapy on non-seasonal depression appears to be controversial. On one hand, some studies support the usefulness and efficacy of this type of therapy [19]. On the other hand, there are also studies expressing doubts about the effect of phototherapy on non-seasonal depression [9]. Nevertheless, most studies suggest that phototherapy could be a valuable treatment method, especially when combined with pharmacological treatment, being mainly considered as an augmentation method for antidepressant medication [26].

Another effect of phototherapy is the improvement in sleep quality. One may question the mechanism behind this, whether it is through the inhibition of melatonin secretion, another neurophysiological mechanism, or simply that improving the patient’s depressive state leads to the automatic amelioration of depressive symptoms such as sleep disturbances [9,19]. There are hypotheses related to the mechanism of action of phototherapy, but they are not fully understood.

Phototherapy has also proven to be beneficial in elderly patients; after phototherapy sessions, the intensity of depression in these patients decreased, and no significant adverse reactions were reported [19,20,36]. This finding may open new avenues in the treatment of depression in older adults. This patient group is the most vulnerable and has limited pharmacological treatment options due to somatic comorbidities, personal medication that may interact with psychiatric medication, and altered metabolism. These factors limit medication options, with some medications being contraindicated or unable to be administered in higher doses. Thus, a complementary and safe method is needed for this patient group, and phototherapy has proven to be effective as an augmentation method for antidepressant treatment and is free of significant adverse reactions [26].

Phototherapy also affects patients with psychiatric comorbidities. For example, in psychosomatic patients, combining occupational therapy with phototherapy has been observed to result in a reduction in depressive and anxious symptoms. This can be objectively demonstrated through psychological scales measuring the severity of these symptoms; after combining these therapies, a decrease in scores was noted, highlighting two significant points: phototherapy is not limited to patients with depression alone, and this type of treatment can be combined with non-pharmacological therapeutic measures, such as psychological ones [23,27].

Another aspect involves patients with depression and borderline personality disorder, where it has been observed that phototherapy, together with antidepressant pharmacological treatment, leads to remission of symptoms, including both depressive symptoms and those specific to borderline personality disorder, such as self-harm tendencies, feelings of emptiness, and impulsivity [26].

Phototherapy, as a non-pharmacological treatment method, can be combined with both pharmacological and psychological methods [29]. Furthermore, the indications for this method could be extended to other psychiatric conditions, such as somatizations and personality disorders [17].

Various colors are used in phototherapy. However, in most cases, color does not influence the antidepressant effect. White, blue, and green light have similar effects [15,16,17]. However, not all types of light have proven to be effective. For example, red light has not shown significant results in treatments [15].

Another aspect is the duration of sessions, with the standard duration being 30 min. Some studies suggest that a session can vary between 30 min and 2 h [15]. The choice of session duration may depend on other factors, such as the intensity of the light used, the number of sessions performed per day, the severity of the patient’s depression, and the patient’s response to previous phototherapy sessions [15,27]. Therefore, depending on these factors, the duration of phototherapy may be adjusted and personalized for each patient to maximize the benefits of the treatment [15].

Although phototherapy is typically administered once a day, some studies suggest that having two sessions per day may be more effective [15,27]. Several practical considerations, such as a faster improvement in mood and better adaptation of the circadian rhythm, can explain this increased efficiency [15]. Additionally, administering two sessions per day allows the patient to receive a higher dosage of light, while breaking up a larger dose can prevent eye irritation that might occur with a single, more intense session [15,30]. Thus, by having more sessions per day, the patient can receive the same total light dose but in a more tolerable manner [15].

Another important aspect is the timing of phototherapy. Depending on the patient’s needs, it can be used both in the morning and evening [9]. For example, conducting sessions in the evening is preferable for patients with insomnia, as phototherapy can improve sleep quality [20]. Conversely, in cases of daytime sleepiness, phototherapy can be used in the morning to reduce this sleepiness by influencing melatonin levels [9,22].

Light intensity varies; even lower light intensities can produce positive effects [15]. The response to phototherapy may differ from patient to patient; some may respond to lower doses, while others require higher doses, with the maximum dose being 10,000 lux [1,6,7]. Other variables affecting the choice of light intensity include the number of sessions per day, the duration of each session, the severity of the depression, and the patient’s ability to tolerate the light [15,24].

Thus, the number of sessions per day, the duration of each session, and the light intensity are adjusted according to the patient’s characteristics, making the application of phototherapy personalized to meet each person’s specific needs [15,24].

Regarding the safety of phototherapy, it proves to be a safe method. Although not entirely free from adverse reactions, these are rare and, when they occur, are transient and do not impact the patient’s long-term quality of life. The literature indicates that contraindications are mainly limited to patients with ophthalmological conditions, but these contraindications are more recommendations for caution rather than absolute contraindications [19,37]. Even patients with ophthalmological conditions can benefit from phototherapy, provided they undergo an ophthalmological consultation before starting the treatment to assess their condition and adjust the phototherapy sessions to prevent discomfort [19]. The lack of absolute contraindications and significant adverse reactions makes phototherapy a safe method, accessible to many patients without being limited by adverse effects or somatic comorbidities.

The present review is limited by the number of studies included. This limitation arose because the literature contains a limited number of studies addressing the effect of phototherapy in unipolar non-seasonal depression. Most studies have explored the impact of phototherapy on seasonal depression. We included studies on seasonal depression for comparative purposes to evaluate the effects of phototherapy in both types of depression. Another limitation is the heterogeneity of studies: the included studies employed different research approaches, studied populations, and measured outcomes, which led to difficulties in interpreting and synthesizing the data. Additionally, there is selective publication of studies; some studies with unfavorable results may not have been published, while studies with positive results may be more frequently published.

Furthermore, the studies were restricted to those published in English, excluding valuable research in other languages that may have contributed new information or relevant data. The studies were conducted over varying periods, reflecting differences in scientific knowledge, technologies, and clinical practices during research. Many included studies were meta-analyses and systematic reviews, some focusing on specific populations or contexts, making it difficult to generalize the findings broadly. Lastly, the analysis omitted unpublished studies, which could offer new data that might either confirm or refute the conclusions drawn from the included studies.

Phototherapy should be considered as a complementary treatment for non-seasonal depression, particularly in elderly patients and those with psychiatric comorbidities, as it is most effective when combined with antidepressants or therapies like occupational therapy. Clinicians should prioritize its use for treatment-resistant depression, psychosomatic disorders, or borderline personality disorder, given its potential benefits. Additionally, since phototherapy improves nocturnal sleep quality and reduces daytime sleepiness, it can be integrated into broader sleep management strategies for depressive patients. Further research is needed to refine its protocols, but clinicians should personalize treatment plans based on individual patient responses and consider phototherapy as a viable adjunctive option.

These practical recommendations emphasize the importance of integrating phototherapy into existing treatment plans for non-seasonal depression, particularly for patients who may benefit the most, such as the elderly and those with psychiatric comorbidities. By combining phototherapy with antidepressants or therapies like occupational therapy, clinicians can enhance its effectiveness, especially in treatment-resistant cases. Its positive effects on sleep suggest that it can also be used to address sleep disturbances commonly associated with depression, improving overall well-being. Since the response to phototherapy may vary among individuals, healthcare providers should tailor its use based on patient needs while further research helps refine optimal protocols and implementation strategies.

## 5. Conclusions

This study highlights the potential role of phototherapy as a supportive treatment for non-seasonal depression, though its effectiveness remains debated. Evidence indicates positive impacts on well-being and symptom relief, especially in elderly patients and those with psychiatric comorbidities like psychosomatic disorders or borderline personality disorder. Phototherapy also benefits sleep by improving nocturnal quality and reducing daytime sleepiness. While phototherapy is used alone for seasonal depression, its effectiveness in non-seasonal cases appears strongest when combined with antidepressants or therapies like occupational therapy, with benefits even in treatment-resistant cases. Standard sessions last 30 min at 4000–10,000 lux in the morning, though research suggests two sessions per day or lower light intensities can also be effective. The safety profile is favorable, with minimal and transient side effects, such as mild agitation and eye irritation, making phototherapy broadly suitable across patient populations, including older adults and those with additional health conditions.

## Figures and Tables

**Table 1:** Analyzed studies.

No.	Authors	Study	Keywords	Main Results
1	Alotaibi MA, Halaki M, Chow CM [15]	A systematic review of light therapy on mood scores in major depressive disorder: light specification, dose, timing and delivery.	Non-seasonal depression, phototherapy, efficacy, lux, duration, white light, blue light, green light, and seasonal depression	Exposure to light for 30 min to 2 h per day, with an intensity between 176 and 10,000 lux, in blue, green, or white light, especially in the morning, has a positive effect on mood.
2	Chang CH, Liu CY, Chen SJ, Tsai HC [16]	Efficacy of light therapy on non-seasonal depression among elderly adults: A systematic review and meta-analysis.	Non-seasonal depression, phototherapy, efficacy, augmentation, adverse effects, lux, duration, white light, blue light, green light, and seasonal depression	Significant efficacy of light therapy in the treatment of geriatric depression.
3	Do A, Li VW, Huang S, Michalak EE, Tam EM, Chakrabarty T, et al. [17]	Blue-light therapy for seasonal and non-seasonal depression: A systematic review and meta-analysis of randomized controlled trials.	Non-seasonal depression, phototherapy, efficacy, adverse effects, lux, duration, white light, blue light, and seasonal depression	The efficacy of blue-light therapy in the treatment of seasonal and non-seasonal MDD remains unproven.
4	Even C, Schroder CM, Friedman S, Rouillon F [18]	Efficacy of light therapy in non-seasonal depression: A systematic review.	Non-seasonal depression, phototherapy, efficacy, duration, and seasonal depression	The efficacy of light therapy in the treatment of and non-seasonal MDD as additional therapy to antidepressant medication.
5	Geoffroy PA, Schroder CM, Reynaud E, Bourgin P [19]	Efficacy of light therapy versus antidepressant drugs, and of the combination versus monotherapy, in major depressive episodes: A systematic review and meta-analysis.	Non-seasonal depression, phototherapy, efficacy, augmentation, adverse effects, lux, duration, white light, blue light, green light, and seasonal depression	Light therapy (LT) and antidepressants (AD) showed no significant differences in effectiveness, but their combination proved superior. Therefore, both LT monotherapy and the combination can be considered as first-line treatments for seasonal and non-seasonal depression.
6	Güdücü F, Çaliyurt O, Vardar E, Tuğlu C, Abay E [20]	Combination therapy using sertraline with sleep deprivation and light therapy compared to sertraline monotherapy for major depressive disorder.	Non-seasonal depression, phototherapy, efficacy, lux, duration, white light, and seasonal depression	Light therapy with sertraline was more effective than sertraline monotherapy for accompanied anxiety in depression.
7	Lee TMC [21]	Phototherapy for SAD: A meta-analytic review.	Phototherapy, efficacy, adverse reactions, lux, duration, and seasonal depression	Phototherapy can be used in typical and atypical depression.
8	Müller MJ, Seifritz E, Hatzinger M, Hemmeter U, Holsboer-Trachsler E [22]	Side effects of adjunct light therapy in patients with major depression.	Non-seasonal depression, phototherapy, bright light therapy, efficacy, augmentation, adverse effects, lux, duration, and seasonal depression	Side effects of phototherapy.
9	Nixon A, Glozier N, Fields K, Wallis R, Biddle D, Chan C, et al. [23]	Improvements in subjective sleep and depression along the course of adjunctive phototherapy.	Non-seasonal depression, phototherapy, lux, duration, blue light, and green light	Effects of bright light are associated with the degree of sleep improvement.
10	Penders TM, Stanciu CN, Schoemann AM, Ninan PT, Bloch R, Saeed SA [24]	Bright light therapy as augmentation of pharmacotherapy for treatment of depression: A systematic review and meta-Analysis.	Non-seasonal depression, bright light therapy, efficacy, augmentation, lux, and seasonal depression	Pooled data analysis confirms that bright light therapy (≥5000 lux for ≥30 min) effectively enhances standard antidepressant treatment for major depressive disorder and non-seasonal bipolar depression.
11	Perera S, Eisen R, Bhatt M, Bhatnagar N, de Souza R, Thabane L, et al. [25]	Light therapy for non-seasonal depression: systematic review and meta-analysis.	Non-seasonal depression, phototherapy, efficacy, lux, duration, white light, blue light, green light, and seasonal depression	Despite the poor overall quality of evidence due to bias and inconsistency, light therapy’s minimal side effects and positive response rates suggest it may be an effective adjunct treatment for non-seasonal depression.
12	Prasko J, Brunovsky M, Latalova K, Grambal A, Raszka M, Vyskocilova J, et al. [26]	Augmentation of antidepressants with bright light therapy in patients with comorbid depression and borderline personality disorder.	Non-seasonal depression, phototherapy, efficacy, augmentation, adverse effects, lux, duration, white light, and seasonal depression	Phototherapy has led to improvements in patients with borderline personality disorder and depression.
13	Pudikov IV, Dorokhov VB [27]	The special physiological importance of the UV-A spectrum for successful phototherapy.	Phototherapy, lux, duration, and seasonal depression	Phototherapy can be successfully used for correcting the state of patients with seasonal depressive disorders.
14	Tayebmanesh L, Saadati N [28]	Effectiveness of integrating quality of life based therapy and phototherapy on emotion regulation, depression, and anxiety in psychosomatic patients of Isfahan City.	Non-seasonal depression, phototherapy, and efficacy	The results indicated that the integration of quality of life-based therapy and phototherapy was effective in improving emotion regulation, depression, and anxiety in psychosomatic patients.
15	Veleva BI, van Bezooijen RL, Chel VGM, Numans ME, Caljouw MAA [29]	Effect of ultraviolet light on mood, depressive disorders and well-being.	Non-seasonal depression, phototherapy, efficacy, lux, duration, white light, green light, and seasonal depression	Six out of seven studies showed that exposure to ultraviolet radiation improved mood, supporting the positive effect of ultraviolet light on mood.
16	Yerevanian BI, Anderson JL, Grota LJ, Bray M [30]	Effects of bright incandescent light on seasonal and non-seasonal major depressive disorder.	Non-seasonal depression, phototherapy, efficacy, adverse effects, lux, white light, and seasonal depression	Phototherapy was ineffective for non-seasonal patients, whose functioning was more impaired than that of seasonal patients even before the trial.

**Table 2:** Study characteristics.

Author	Study	Country	Year	Study Design	Sample Size
Alotaibi MA, Ha-laki M, Chow CM [15]	A systematic review of light therapy on mood scores in major depressive disorder: light specification, dose, timing and delivery	Australia	2016	Systematic review	24 studies
Chang CH, Liu CY, Chen SJ, Tsai HC [16]	Efficacy of light therapy on non-seasonal depression among elderly adults: A systematic review and meta-analysis	China	2018	Systematic review and meta-analysis	8 studies
Do A, Li VW, Huang S, Michalak EE, Tam EM, Chakrabarty T, et al. [17]	Blue-light therapy for seasonal and non-seasonal depression: A systematic review and meta-analysis of randomized controlled trials	Canada	2022	Systematic Review and meta-analysis of randomized controlled trials	9 studies
Even C, Schroder CM, Friedman S, Rouillon F [18]	Efficacy of light therapy in non-seasonal depression: A systematic review	France	2008	Systematic review	15 studies
Geoffroy PA, Schroder CM, Reynaud E, Bourgin P [19]	Efficacy of light therapy versus antidepressant drugs, and of combination versus monotherapy, in major depressive episodes: A systematic review and meta-analysis	France	2019	Systematic review and meta-analysis	7 studies
Güdücü F, Çaliyurt O, Vardar E, Tuğlu C, Abay E [20]	Combination therapy using sertraline with sleep deprivation and light therapy compared to sertraline monotherapy for major depressive disorder	Turkey	2005	Original study	37 patients
Lee TMC [21]	Phototherapy for SAD: A meta-analytic review	Canada	1995	Systematic review and meta-analysis	Studies
Müller MJ, Seifritz E, Hatzinger M, Hemmeter U, Holsboer-Trachsler E [22]	Side effects of adjunct light therapy in patients with major depression	Germany	1997	Original study	42 patients
Nixon A, Glozier N, Fields K, Wallis R, Biddle D, Chan C, et al. [23]	Improvements in subjective sleep and depression along the course of adjunctive phototherapy	Canada	2017	Original study	22 patients
Penders TM, Stan-ciu CN, Schoemann AM, Ninan PT, Bloch R, Saeed SA [24]	Bright light therapy as augmentation of pharmacotherapy for treatment of depression: A systematic review and meta-analysis	USA	2016	Systematic review and meta-analysis	10 studies
Perera S, Eisen R, Bhatt M, Bhatnagar N, de Souza R, Thabane L, et al. [25]	Light therapy for non-seasonal depression: systematic review and meta-analysis	UK	2016	Systematic review and meta-analysis	20 randomized controlled trials
Prasko J, Brunovsky M, Latalova K, Grambal A, Raszka M, Vyskocilova J, et al. [26]	Augmentation of antidepressants with bright light therapy in patients with comorbid depression and borderline personality disorder	Czech Republic	2010	Original study	13 female patients
Pudikov IV, Dorokhov VB [27]	The special physiological importance of the UV-A spectrum for successful phototherapy	Russia	2012	Original study	24 patients
Tayebmanesh L, Saadati N [28]	Effectiveness of integrating quality of life based therapy and phototherapy on emotion regulation, depression, and anxiety in psychosomatic patients of Isfahan City	Iran	2023	Original study	30 patients
Veleva BI, van Be-zooijen RL, Chel VGM, Numans ME, Caljouw MAA [29]	Effect of ultraviolet light on mood, depressive disorders and well-being	The Netherlands	2018	Systematic review	7 studies
Yerevanian BI, Anderson JL, Grota LJ, Bray M [30]	Effects of bright incandescent light on seasonal and non-seasonal major de-pressive disorder	The Netherlands	1986	Original study	43 patients

## Data Availability

Data are contained within this article.
